# A Fast and Simple Contact Printing Approach to Generate 2D Protein Nanopatterns

**DOI:** 10.3389/fchem.2018.00655

**Published:** 2019-01-24

**Authors:** Marco Lindner, Aliz Tresztenyak, Gergö Fülöp, Wiebke Jahr, Adrian Prinz, Iris Prinz, Johann G. Danzl, Gerhard J. Schütz, Eva Sevcsik

**Affiliations:** ^1^Institute of Applied Physics, TU Wien, Vienna, Austria; ^2^Stratec Consumables GmbH, Anif, Austria; ^3^Institute of Science and Technology Austria, Klosterneuburg, Austria

**Keywords:** contact printing, protein patterning, nanopatterns, nanofabrication, super-resolution fluorescence microscopy, STED microscopy

## Abstract

Protein micropatterning has become an important tool for many biomedical applications as well as in academic research. Current techniques that allow to reduce the feature size of patterns below 1 μm are, however, often costly and require sophisticated equipment. We present here a straightforward and convenient method to generate highly condensed nanopatterns of proteins without the need for clean room facilities or expensive equipment. Our approach is based on nanocontact printing and allows for the fabrication of protein patterns with feature sizes of 80 nm and periodicities down to 140 nm. This was made possible by the use of the material X-poly(dimethylsiloxane) (X-PDMS) in a two-layer stamp layout for protein printing. In a proof of principle, different proteins at various scales were printed and the pattern quality was evaluated by atomic force microscopy (AFM) and super-resolution fluorescence microscopy.

## Introduction

In recent years, surfaces featuring micropatterns of biomolecules, particularly proteins, have seen a surge of interest. They have found multiple applications in biomedical research such as microarrays (Macbeath and Schreiber, 2000; Allison et al., 2006; Wingren and Borrebaeck, 2007), proteomics (Haab, 2005; Wingren and Borrebaeck, 2008), and biomimetic sensors (Mujahid et al., 2013). Furthermore, cell-scale microstructured biointerfaces have been used to manipulate cell shape and organization to study endocytosis (Tan et al., 2015), cell polarization and proliferation (Chen et al., 1997; Théry, 2010), host-pathogen interactions (March et al., 2015) as well as stem cell differentiation (Sykova and Forostyak, 2013; Castaño et al., 2014). On a smaller scale, micropatterned surfaces have been applied to influence the protein distribution within living cells to address several cell biological questions such as plasma membrane organization (Sevcsik et al., 2015; Fülöp et al., 2018), dynamics of cytokine receptor signaling (Löchte et al., 2014), T cell signaling (Mossman et al., 2005), protein-protein interactions (Lanzerstorfer et al., 2014; Dirscherl et al., 2018), phagocytosis (Freeman et al., 2016), and cell adhesion (Schvartzman et al., 2011; Coyer et al., 2012). Different techniques have been developed for fabricating patterned surfaces which cater to the demands of the respective applications.

One family of techniques is based on indirect deposition of proteins; the most prominent of these are photolithography (Christman et al., 2006; Lenci et al., 2011) and laser microablation (Nicolau et al., 2010), where the minimum feature sizes are set by the diffraction limit of light. This restriction can be overcome by colloidal lithography (Kristensen et al., 2013), di-block copolymer micelle nanolithography (Thelen et al., 2007; Lohmüller et al., 2011) or di-block copolymer self-assembly (Hortigüela et al., 2017). A different approach of indirect deposition employed electropolymerization to functionalize gold micro- and nanoelectrodes with proteins (Della Pia et al., 2014). Methods based on direct deposition of proteins include maskless projection lithography (Waldbaur et al., 2012), microfluidic patterning (Delamarche et al., 1998; Alom Ruiz and Chen, 2007), and contact-based printing. Here, one of the most convenient, cost-efficient, and widely used methods has been microcontact printing (μCP) with poly(dimethylsiloxane) (PDMS) stamps (Wilbur et al., 1994; Bernard et al., 2000). PDMS has several properties that made it a perfect choice for a stamp material: it is elastomeric and chemically inert, it molds with high fidelity and is, due to its low surface energy, easily removed from the mold after curing as well as from the substrate after printing (Alom Ruiz and Chen, 2007).

While several convenient methods exist today to create microstructured surfaces, the fabrication of patterns with a feature size below 1 μm is still challenging and often requires trade-offs between speed, biocompatibility, cost, versatility, and experimental complexity. Easy availability of high-performance nano-biointerfaces would open up completely new vistas for proteomics and cell research: in case of protein biochips, the total chip surface and hence the required amount of sample material for analysis could be massively reduced; using nano-biointerfaces as substrates for living cells allows for investigating the influence of protein organization at the nanoscale on signaling (Deeg et al., 2013; Shaw et al., 2014; Cai et al., 2018), adhesion (Arnold et al., 2004; Cavalcanti-Adam et al., 2007), or cell differentiation (Wang et al., 2015). Additionally, nm-sized structures fit capabilities of state-of-the-art super-resolution microscopy readout technologies (Sahl et al., 2017). For this purpose, dip-pen nanolithography (Lee et al., 2002; Chai et al., 2011), electron beam lithography (Zhang et al., 2007), or STED lithography (Harke et al., 2012; Wiesbauer et al., 2013; Fischer et al., 2015) have the advantage of a high resolution and full freedom of choice regarding the created protein patterns, but are typically slow and require complex procedures and equipment. On the other hand, μCP has seen widespread adoption due to its simplicity and good performance. However, printing with PDMS stamps typically results in sagging of the interspaces or pairing for features significantly below 1 μm (Schmid and Michel, 2000; Verschuuren, 2009; Kaufmann and Ravoo, 2010). Therefore, many creative methods were developed to circumvent the limitations of PDMS as a stamp material for nanocontact printing.

One successful approach to create features below 100 nm made use of silicone stamps with pyramidical features (Li et al., 2003; Filipponi et al., 2016). A drawback of this stamp architecture is that only periodicities in the micrometer range can be realized. A different strategy to improve resolution is to increase the Young's modulus of the stamp material. This, however, can entail changes of the material properties compared to PDMS with respect to surface energy, as well as the necessity for complex procedures in stamp production. Thus, the fundamental advantages of silicone such as ease-of-use, compliant contact, and low surface energy, are often abrogated. For instance, polyolefin plastomers foils can yield imprints of superior quality in the sub-micrometer range compared to PDMS, however, the stamp development requires hot embossing (Schwaab et al., 2013). Therefore, global flatness is hard to achieve after demolding and requires equipment with a high cost of ownership especially for larger stamps (Shan et al., 2008). Furthermore, the material is not gas permeable which increases the appearance of trapped air bubbles, particularly in a manual printing procedure. Another example is the use of a PDMS derivative with increased Young's modulus of up to 9 MPa for printing proteins with a periodicity down to 210 nm (Renault et al., 2003). The density of proteins that could be achieved with stamps featuring 100 nm pillars, however, was very low and imprints exhibited a number of defects.

Here, we present a nanocontact printing approach to fabricate highly condensed 2D nanopatterns of proteins that has all the advantages of silicone-based printing while avoiding its drawbacks. This is made possible by utilizing the novel stamp material X-PDMS in a 2-layer stamp architecture which has been developed and to our knowledge so far only been applied for substrate conformal imprint lithography (SCIL) (Verschuuren et al., 2017). For SCIL, the authors developed a flexible silicone stamp consisting of a high modulus silicone (X-PDMS) on the feature side and a soft low modulus silicone (PDMS) as a backplane. The different material properties of the two silicones are a consequence of the polymer architecture: PDMS cures into a two-dimensional network with a maximum Young's modulus of 2.5 MPa, while the comparatively high stiffness of X-PDMS is achieved by the addition of tetrafunctional siloxanes, which enable polymerization in three dimensions and thus increase the Young's modulus to up to 85 MPa (Verschuuren, 2009).

Thus, the 2-layer stamp combines the advantages of two worlds: allowing features and a periodicity down to the nanometer range and having a long lifetime while still ensuring conformal contact between non-perfect surfaces over large areas and being easy to fabricate. We exemplify our approach by creating a nanostructured antibody surface with feature sizes of 80 nm on a biocompatible background and evaluating the created protein patterns by atomic force microscopy (AFM) and stimulated emission depletion (STED) microscopy.

## Materials and Methods

### Master Design

Masters for X-PDMS casting of stamps with 300 nm feature size as well as pillars with 80 nm diameter were fabricated by phase transition mastering (PTM), a process developed and typically applied for mastering of Blu-ray disks (Osato, 2003; Chang et al., 2011). PTM was performed with a PTR-3000 master device modified for writing patterns different to Blu-ray designs. PTM allows relative freedom regarding the design of features, but has limits set, among others, by size of the laser spot used for writing the patterns. For PTM, first, a layer of amorphous silicon (~100 nm) was sputtered on an 8 inch silicon wafer followed by a second layer of imperfect oxides of tungsten and molybdenum (phase transition material, ~100 nm) (Chang et al., 2011). Irradiation with a 405 nm laser changed the state of the phase transition material from amorphous to polycrystalline, resulting in radial patterns produced by constant spinning of the wafer. The patterns were then developed using tetramethylammonium hydroxide wet chemistry. Next, masters were plated with nickel to ensure a smooth surface (Ra < 1 nm). During a resting period of 1 week, nickel was allowed to form an inert oxide layer to prevent abrasion during stamp casting. By carefully adjusting the sputtering parameters, the diameter of the wells in the master could be decreased down to 80 nm width (a design that will result in pillars of this diameter). The available tools, however, did not allow to fabricate a master with pillar feature sizes smaller than 300 nm and a period significantly smaller than 600 nm. The master for casting stamps featuring 80 nm sized wells with a periodicity of 140 nm was thus bought from Eulitha, Switzerland.

### Stamp Fabrication

X-PDMS stamps were prepared following a protocol described in Verschuuren (2009). Commercially available X-PDMS (SCIL Nanoimprint solutions, Philips, Netherlands) consists of two components, A and B, which are of proprietary composition. The basis, however, has been published (Verschuuren, 2009): component A consists of a mixture of vinyl siloxanes including 3D-branching Q-siloxanes, a platinum catalyst (platinum di-vinyl-tetra-methyl-di-siloxane), and a moderator (1,3,5,7-tetra-vinyl-1,3,5,7-tetra-methyl-cyclo-tetra-siloxane); component B consists of hybrid linear siloxanes, which are crosslinked upon mixing with component A. To achieve the highest Young's modulus of ~85 MPa (Verschuuren, 2009), components A and B were mixed in a ratio of 0.325:1 and subsequently degassed in a centrifuge with 2000 rpm for 3 min. Three grams of the mixture were poured on the wafer, spin coated at 2,000 rpm for 30 s, and pre-cured for several minutes at 70°C. Next, 3 g of Intermediate Layer (SCIL Nanoimprint solutions, Philips, Netherlands) was added, spin coated, and the stack was cured for 10 days at 70°C. Finally, a 2 mm layer of PDMS was added and cured. The stamp surface was thoroughly washed with ethanol, iso-propanol, and deionized water (DIW) to remove residual monomers. The quality of the stamps was evaluated by scanning electron microscopy (SEM) in secondary electrons mode on a Hitachi S-4000 with EMI compensation system MK3 (Integrated Dynamics Engineering, Germany). For SEM, small areas of the stamps were sputtered with a gold layer of ~20–30 nm.

### Nanocontact Printing

Glass coverslips (#1.5, 24 × 60 mm; Menzel®, Fisher Scientific, USA) were coated with Mix&Go™ Biosensor (Anteo Diagnostics Ltd, Australia) following the manufacturer's protocol. Briefly, a glass coverslip was cleaned thoroughly in an ultrasonic bath with acetone and DIW. Directly afterwards, 100 μl of Mix&Go® fluid was pipetted onto the coverslip, incubated for 45 min, washed with DIW and dried in a stream of nitrogen.

Nanocontact printing was performed essentially following a previously published protocol (Schütz et al., 2017). Briefly, stamps were incubated with the desired protein (100 μg/ml bovine serum albumin, BSA) or fibronectin (FNT, both from Sigma-Aldrich, USA) in phosphate-buffered saline (PBS, Sigma-Aldrich, USA) for 15 min, rinsed thoroughly with distilled water, and dried in a N_2_ flow. After drying, the stamp was placed on a coated coverslip, pressed slightly to ensure good contact and incubated for 15 min at room temperature. After removal of the stamp, patterns were measured immediately or stored at 4°C in UniMailers® Slide Mailers (VWR International GmbH, Austria) sealed with parafilm. Before measurements, the UniMailers were warmed to room temperature before opening to avoid condensation on the slides. Before and after each use, stamps were washed with ethanol, iso-propanol, and DIW to remove dust particles and residual proteins.

### AFM Characterization of Nanopatterned Surfaces

Atomic force microscopy (AFM) was performed on a Dimension Edge AFM (Bruker S.A.S., France) in tapping mode. OTESPA-R3 (Bruker S.A.S., France) cantilevers with a spring constant of ~26 N/m were used for imaging. AFM image analysis was performed in Gwyddion v2.49. Mainly, the correction algorithms “remove polynomial background,” “align rows using various methods,” and “correct horizontal scars (strokes)” were applied for leveling. Background subtraction was performed by taking the signal of the glass coverslip not covered by protein as a reference. For quality assessment of protein transfer by printing, AFM images were converted to 16 Bit grayscale images and further analyzed in ImageJ. For this, regular arrays were selected within the printed patterns (Figure S1) corresponding to either regions with (“ON”) or without (“OFF”) stamp-surface contact. The mean gray values per pixel for each region, I_ON_ and I_OFF_, were used to calculate the contrast $C=\frac{I_{ON}-I_{OFF}}{I_{ON}}$. In AFM image analysis, care was taken to perform the background subtraction the same way in all images to make contrast data comparable. Note though that contrast values are only intended as a means of relative comparison between samples and are no absolute indicator of pattern quality.

### STED Microscopy

For STED microscopy, goat anti-rabbit IgG antibody conjugated with STAR RED (Abberior, Germany) was diluted 1:50 in PBS to a final concentration of ~20 μg/ml. A Secure-Seal® hybridization chamber (Grace Biolabs, USA) was placed on top of the pattern and filled with 50 μl of the antibody solution, incubated for 15 min and washed with 1 ml of PBS to remove unbound antibody.

STED microscopy was performed on an inverted commercial STED microscope (Abberior Instruments, Germany) with pulsed excitation and STED lasers. A 640 nm laser was used for excitation and a 775 nm laser for stimulated emission. An oil immersion objective with numerical aperture 1.4 (UPLSAPO 100XO, Olympus, Japan) was used for imaging. The fluorescence signal was collected in a confocal arrangement with a single photon counting avalanche photodiode using a 685/70 nm bandpass filter. The pulse repetition rate was 40 MHz and fluorescence detection was time-gated. The imaging parameters used for acquiring STED images of W80 patterns were 40 μs dwell time, 41 μW excitation laser power and 80 mW STED laser power. The corresponding confocal images were recorded with 50 μs dwell time and 2 μW excitation laser power. For both STED and confocal images, a pixel size of 10 nm, a pinhole of 0.9 airy units and 3 line accumulations were used. The imaging parameters for acquisition of the STED images of W300 patterns were 40 μs dwell time, 84 μW excitation laser power and 94 mW STED laser power. The corresponding confocal images were recorded with 50 μs dwell time and 12 μW excitation laser power. For both STED and confocal images, a pixel size of 20 nm, a pinhole of 1.0 airy units and 3 line accumulations were used. The power values refer to the power at the back aperture of the objective lens.

## Results and Discussion

**Figure 1:**
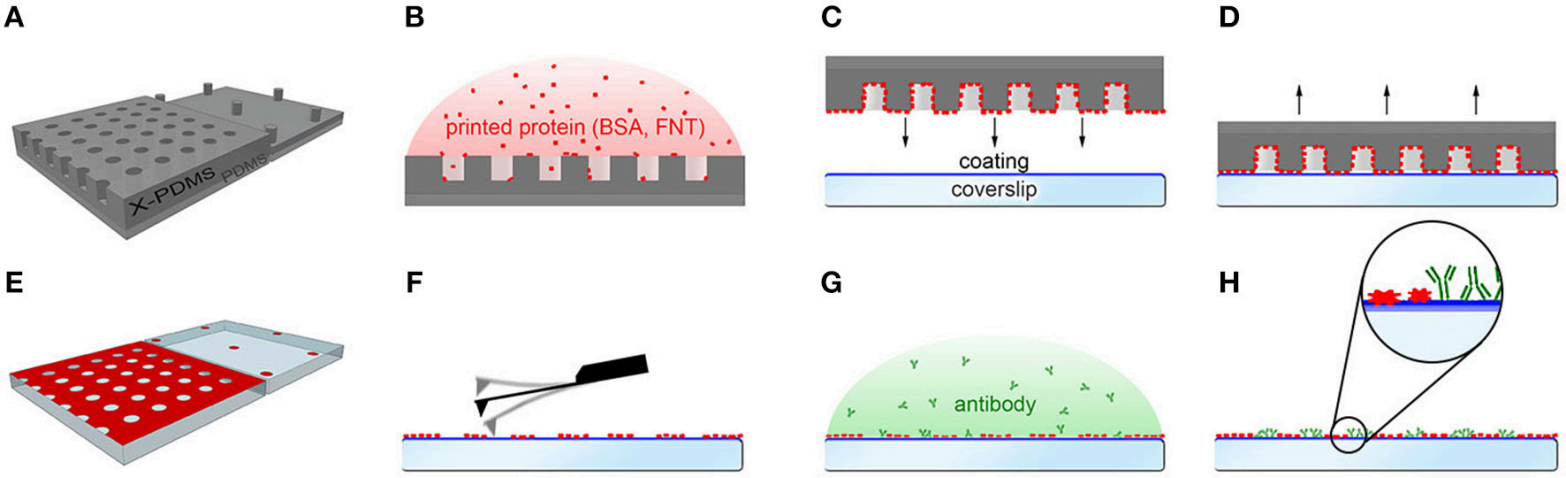
Illustration of the nanocontact printing procedure. **(A)** Sketch of a stamp featuring a well (left) or pillar (right) layout. **(B)** The protein solution is incubated on the X-PDMS/PDMS stamp. **(C–E)** After washing and drying, the stamp is brought into contact with a coverslip coated with MixandGo® Biosensor. The stamp is pressed onto the coverslip, incubated and removed, leaving the protein pattern on the coverslip. **(F)** The quality of the imprint is determined by AFM imaging. **(G,H)** After washing, the antibody is added to fill the interspaces, incubated and rinsed with water to remove unbound antibody.

Our goal was to produce a patterned surface consisting of nanometer-sized antibody dots and a biocompatible background by nanocontact printing. Two different strategies can, in principle, be used to achieve such a surface architecture: (a) printing of an antibody (or a biofunctional protein such as streptavidin) using stamps featuring a pillar profile followed by backfilling with a protein for surface passivation, or (b) printing of a background protein using stamps featuring a well profile and backfilling with antibody or streptavidin. In view of versatility of the produced patterns as well as long-term storability, we chose to adopt the latter approach to print BSA, a protein routinely used for surface passivation. The principle of surface preparation is illustrated in Figure 1.

**Figure 2:**
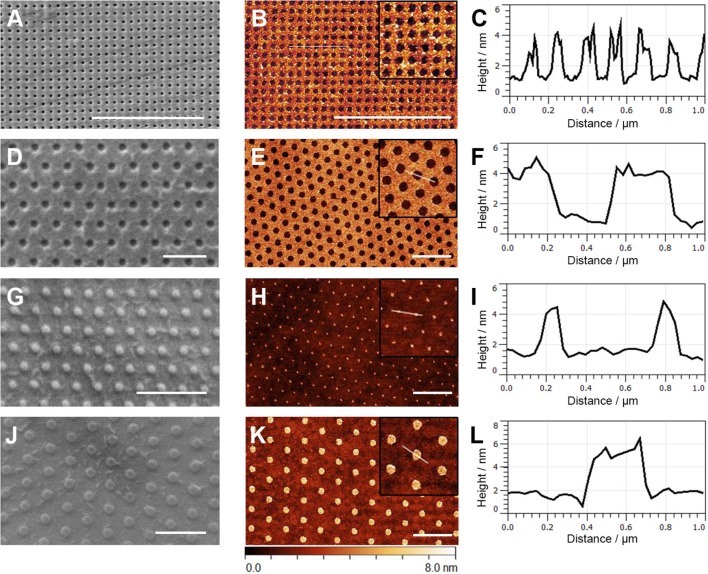
Images of the used stamps and printed BSA patterns. The first column shows SEM images of the different stamps used in this study. AFM images of printed BSA are shown in the middle column; zoom-ins are shown in the insets. The white lines correspond to the line profiles shown in the third column. The rows show data of the different stamp layouts as follows: **(A–C)** W80, **(D–F)** W300, **(G–I)** P80, **(J–L)** P300. Scale bar is 2 μm in all images.

Different strategies for surface preparation for protein printing have been proposed: plasma cleaning (Ricoult et al., 2014; MacNearney et al., 2016), epoxy functionalization (Sevcsik et al., 2015), streptavidin-coating (Lanzerstorfer et al., 2014) as well as no further treatment of the glass substrate (Dirscherl et al., 2018). To achieve not only high imprint quality but also good long-term storability of the printed nanopatterns, we chose to functionalize the glass coverslips with Mix&Go® Biosensor, a polymer metal ion coating that does not react with water and allows strong attachment of proteins via avidity binding. The first tested stamp layout featured wells of 80 nm diameter and 140 nm periodicity (W80). Table 1 summarizes the features of the different stamp layouts used in this study. A SEM image of the W80 stamp is shown in Figure 2A. AFM images of the BSA patterns printed with W80 revealed defect-free imprints, with an average height of the printed protein of ~3 nm corresponding to a monolayer of BSA (Figures 2B,C).

**Table 1:** Features of the used stamps.

**Stamp**	**Type**	**Diameter (nm)**	**Period (nm)**	**Depth (nm)**
W80	Wells	80	140	100
W300	Wells	300	600	100
P80	Pillars	80	600	100
P300	Pillars	300	600	100

For comparison, we printed patterns with a larger feature size of 300 nm and a periodicity of 600 nm (W300, Figures 2D–F). To quantitatively assess and compare imprint quality, the contrast *C* between “ON” regions (where protein transfer should occur) and “OFF” regions (where no protein transfer should occur) was determined; contrast values are summarized in Table 2. Printing of BSA using the W80 stamps was reproducible with a mean contrast of 0.61, which was lower than for stamps with the W300 layout (*C* = 0.78). There are several possible explanations for this: first, out of the 10 imprints analyzed for the W80 layout, 2 exhibited contrast values below 0.4. Such outliers were not observed for the W300 layout; they may be a consequence of the manual printing process. Second, AFM imaging artifacts can compromise contrast values. For example, edge effects caused by proteins being dragged into the “OFF” areas are more pronounced with decreasing feature size and lead to an overestimation of the height in “OFF” areas, resulting in an overall decreased contrast.

**Table 2:** Quality assessment of protein patterns.

**Stamp layout**	**Protein**	***C***	***n***	**Comments**
W80	BSA	0.61 ± 0.14	10	2 samples with *C* < 0.40
W300	BSA	0.78 ± 0.08	12	
W 300	BSA	0.79 ± 0.02	3	3 different ROIs within one 6″ imprint
W80	BSA	0.58 ± 0.07	3	after 50 prints
W80	BSA	0.53 ± 0.03	3	after 17 days at 4°C
W80	FNT	0.26 ± 0.11	9	0 samples with *C* > 0.50
W300	FNT	0.67 ± 0.05	14	
P80	BSA	0.13 ± 0.28	6	1 sample with *C* > 0.75
P300	BSA	0.72 ± 0.02	7	
P80	FNT	0.24 ± 0.34	9	2 samples with *C* > 0.75
P300	FNT	0.78 ± 0.02	6	
W80	BSA/antibody	0.55 ± 0.04	3	*C* from STED images
W300	BSA/antibody	0.77 ± 0.03	3	*C* from STED images

*n indicates the number of imprint replicates. Mean contrast values ± SD are given*.

In contact printing, protein deposition does not depend on the coherence of a light source or other long-range effects limiting the size of the patterned area. The size limit for a patterned area is thus set, in principle, by the size of the master. While W80 and P80 masters were 30 × 30 mm, W300 and P300 masters were considerably larger (6 × 6 inches). Even with stamps produced from the latter, pattern quality was generally high over the whole printed area (Table 2). In a manual printing process, a practical limitation is set by the dexterity of the experimenter, since the likelihood of trapping air bubbles between stamp and substrate increases with the stamp size.

The next step was to check whether reuse of the stamps would decrease the imprint quality. Even after 50 prints, however, the contrast of the W80 imprints did not deteriorate (Figure S2A). Another important parameter is the storability of the printed patterns. After 17 days of storage at 4°C, we observed a small decrease in pattern contrast (Figure S2B).

For live cell applications of nanopatterned surfaces, it is sometimes beneficial to promote cell adhesion in the areas next to functionalized dots. In microcontact printing, fibronectin has often been used for this task (Shen et al., 2008). Printing of fibronectin using the W80 stamp yielded patterns of poor contrast (*C* = 0.21) and ring-like features (Figure S3A). In comparison, the imprint qualities obtained with the W300 stamp layout were of much better quality, similar to the ones obtained with BSA (Figure S3B). The poor performance of fibronectin on the W80 stamps is most likely a consequence of the size and properties of the fibronectin molecules: in a compact conformation, the molecular dimensions of fibronectin are ~9 × 16 nm (Koteliansky et al., 1981), while the stretched molecule can reach lengths of up to 160 nm (Erikson et al., 1981). It is thus possible that fibronectin partially covers the 80 nm wells thereby leading to a loss of resolution. Our results indicate though that fibronectin can be used for printing structures with a well layout of and above 300 nm feature size.

In some cases, it may not be feasible to print a background protein and fill with a functional protein. We thus tested the X-PDMS/PDMS stamp architecture for stamps featuring 80 nm pillars (P80, Figures 2G–I) and compared their performance with 300 nm pillars (P300, Figures 2J–L). The performance of the P300 stamps was similar to the W300 ones, however, the mean contrast of the P80 patterns was markedly decreased compared to W80. Closer inspection of the data revealed that while 5 out of 6 produced P80 patterns showed very poor contrast (*C* < 0.1), one imprint was of high quality (*C* = 0.75; shown in Figures 2H,I). This heterogeneity in the performance of the P80 stamps may originate from the manual printing process. Although AFM images did not indicate permanent damage to the stamps after use, it is conceivable that excessive pressure during printing results in a reversible collapse of the rather soft pillars leading to a loss of contrast in the printed pattern. This may be avoided by controlling the pressure during printing by using e.g., a SCIL tool. Interestingly, printing of P80 fibronectin patterns yielded similar results compared to BSA: 2 out of 9 imprints were of high quality (*C* > 0.75, Figure S4), while for the remainder printing was not successful. This suggests that, with controlled pressure, robust printing of 80 nm features of fibronectin may be feasible.

**Figure 3:**
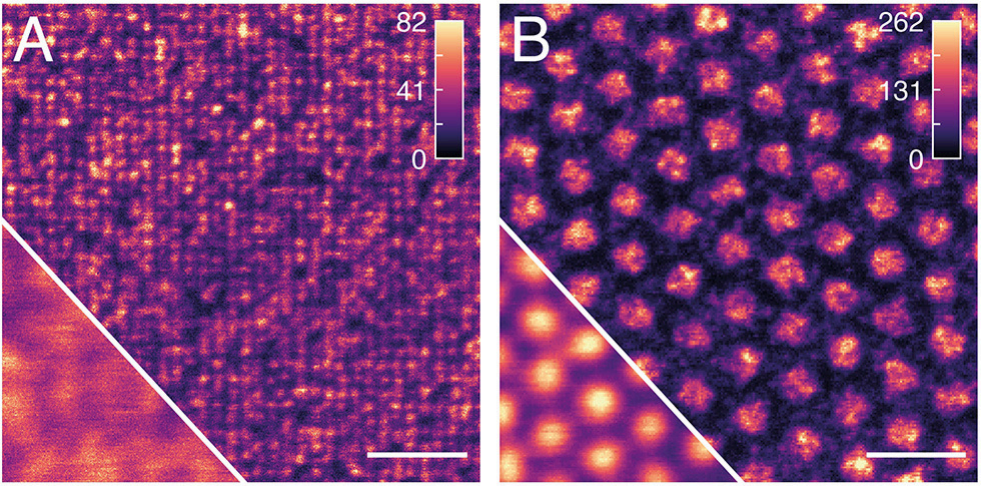
Evaluation of antibody nanopatterns with STED microscopy. Representative areas of **(A)** W80 and **(B)** W300 BSA patterns backfilled with Abberior STAR RED labeled antibody were imaged with STED microscopy. The corresponding confocal images are shown in the bottom left corners. The color map shows the number of detected photons. Scalebar is 1 μm.

We decided to continue our work using W80 BSA patterns. The next step in the process of surface preparation was backfilling with a functional protein. The Mix&Go® Biosensor we used to activate the coverslips for protein attachment was specifically designed to preserve the functionality of antibodies (Ooi et al., 2014). Hence, fluorescently labeled antibody was directly added to coverslips featuring W80 BSA patterns. Since the feature sizes of the nanopatterns are below the diffraction limit of light, conventional fluorescence microscopy cannot be used for quality assessment. We thus employed STED microscopy to visualize the produced antibody nanopatterns. Both the W80 and the W300 patterns are clearly visible in the STED images, while only the W300 features are discernible in the confocal images (Figure 3). Surfaces featuring W300 patterns appear more homogeneous and with less defects than surfaces with W80 patterns. Particularly, in the W80 patterns, some dots seem to be devoid of or only weakly populated by fluorophores, while some dots seem excessively bright. The contrast values determined for W80 and W300 antibody patterns were 0.52 and 0.77, respectively.

Part of the difference between W80 and W300 patterns may be due to a lower contrast already present in the W80 BSA patterns. Several factors can reduce the detected dot brightness and thus the contrast: low degree of labeling of the antibodies, fluorophores lost due to bleaching by either the excitation laser or the high-intensity STED laser, fluorophores that are non-functional to begin with as well as a photon detection efficiency of ~70%. Considering the size of an antibody (10 × 15 nm), ~20–30 antibodies can maximally be accommodated in one well with 80 nm diameter. Due to the stochastic distribution of antibodies on the surface, it is possible that the number of fluorophores and thus detected photons differs significantly between individual dots produced with the W80 stamps. With feature sizes of 300 nm, this will lead to brightness heterogeneities within individual antibody dots (as apparent from Figure 3B), whereas with 80 nm features, heterogeneities between dots will dominate.

## Conclusion

We present here a nanocontact printing approach to generate nanopatterned protein surfaces that strikes a balance between resolution, simplicity, and speed, and validate our method on the example of 80 nm sized antibody dots on a BSA background. Nanocontact printing with X-PDMS stamps on a polymer metal ion coated substrate neither requires a clean room facility nor cost-intensive equipment but allows the fabrication of reproducible highly condensed 2D protein patterns on the nanoscale in a standard lab environment. For feature sizes of 80 nm, we found that stamps with a well layout produced high-contrast imprints with much higher fidelity than pillar stamps. The quality of the printed protein patterns was consistent and robust over large areas, as assessed with AFM and STED microscopy. We showed that the stamps can be reused many times which further reduces the fabrication time of nanopatterned surfaces. In addition, the signal-to-noise ratio of the produced patterns is high enough to support super-resolution microscopy. While all experiments were performed manually, we believe that the contrast as well as the success rate of the imprints could be further improved with a printing tool similar to those used for SCIL. Even with standard lab equipment, the approach presented here is well-suited for a multitude of applications in research laboratories, such as cell adhesion and protein interaction studies but may also prove particularly useful for printing large scale nanoarrays for biosensing or drug discovery. When combined with microfluidics for depositing different capture proteins in parallel, the well layout presented here even allows the fabrication of multi-protein patterns on a single biochip surface.

## Author Contributions

ML, IP, GS, and ES conceived and designed the experiments. ML, AT, and GF performed the experiments and analyzed the data. WJ and JD designed, performed, and analyzed the STED microscopy experiments. ML and AP designed and performed PTM mastering. ML and ES wrote the paper, while all other authors edited and revised the manuscript.

### Conflict of Interest Statement

The authors declare that the research was conducted in the absence of any commercial or financial relationships that could be construed as a potential conflict of interest.
