# Supplementary material for: A Fast and Simple Contact Printing Approach to Generate 2D Protein Nanopatterns
Source: Front Chem. 2019 Jan 24;6:655. doi: 10.3389/fchem.2018.00655 (PMC6353799; doi:10.3389/fchem.2018.00655)
Supplement: Supplementary file 1 [file Data_Sheet_1.PDF]

Supplementary Figures

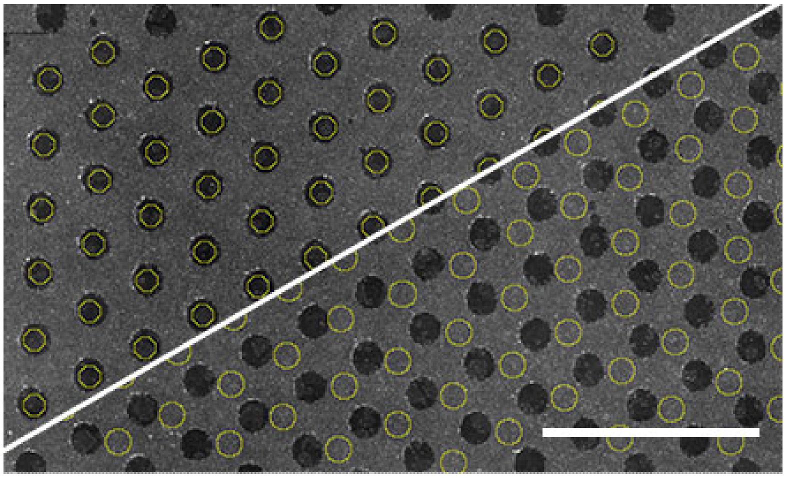

Figure S1. Selection of "ON" and "OFF" areas for contrast determination. "OFF" regions in W80 and W300 patterns were selected by defining repetitive circular features in the pattern image (upper left). The size of the selected features was smaller than the actual well features to avoid edge effects. "ON" regions were selected in a similar fashion (bottom right). A W300 BSA pattern image is shown, scale bar is 2 μm.

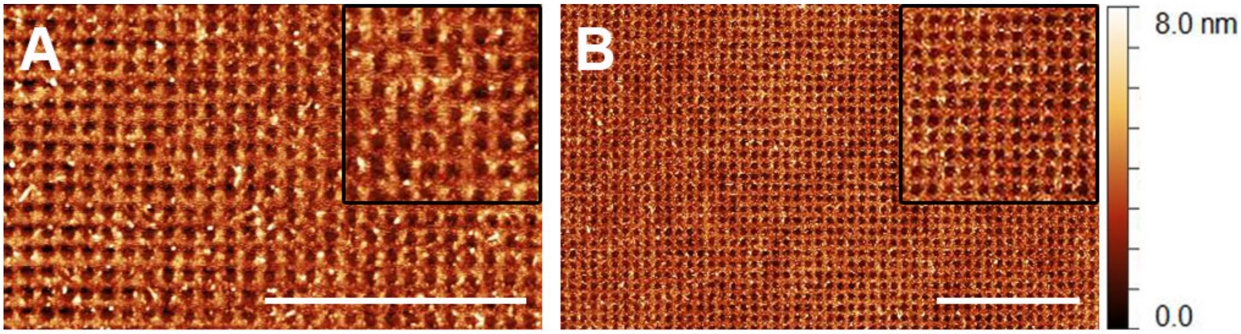

Figure S2. Reuse of W80 stamps and storability of printed BSA patterns. (A) A W80 stamp was used for printing BSA and subsequently washed as described in the Methods section. This was repeated 50 times; the AFM image shows the BSA patterns obtained with the 50th imprint. **(B)** AFM image of a W80 BSA pattern after storage at 4°C for 17 days. Scale bar is 2 μm.

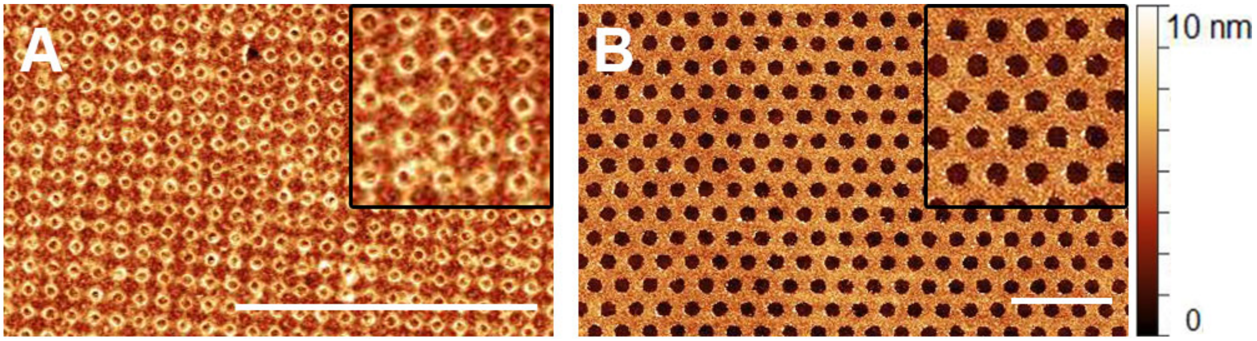

Figure S3. AFM images of fibronectin patterns in a well layout. (A) W80 FNT patterns exhibit ring-like features at the well edges. **(B)** W300 FNT patterns. Zoom-ins are shown in the insets. Scale bar is 2 μm.

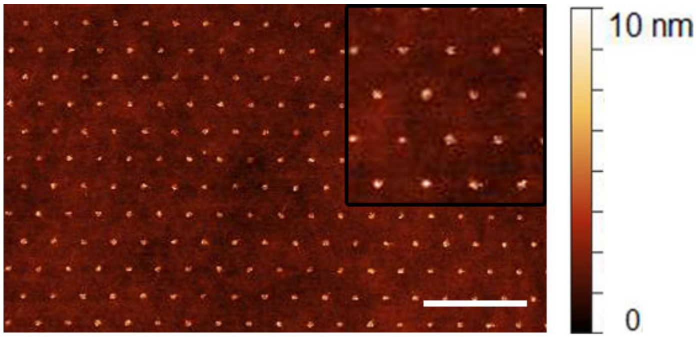

Figure S4. AFM image of P80 fibronectin patterns. A zoom-ins is shown in the inset. Scale bar is 2 μm.
